# Focused Assessment with Sonography in Trauma (FAST) Exam Bedside Evaluation and Validation for Trauma Patients in the Emergency Department

**DOI:** 10.51894/001c.144144

**Published:** 2025-09-30

**Authors:** Andrew Vierra

**Affiliations:** 1 Department of Emergency Medicine McLaren Oakland

## 44

### INTRODUCTION

The Focused Assessment with Sonography in Trauma (FAST) exam has cemented itself as an adjunct to the Advanced Trauma Life Support (ATLS) protocol.  The FAST exam is typically completed at bedside via a large ultrasound machine or an approved handheld device by the surgical team or emergency medicine physician.  At McLaren Oakland Hospital, the FAST exam is required to be uploaded into the Picture Archiving and Communication System (PACS) for verification and validation by the radiologist for ACS level II trauma certification. 

### AIMS/OBJECTIVES

A 3-phase Plan-Do-Study-Act quality improvement (PDSA QI) project was instituted to develop, implement, and assess the utility of a standardized Emergency Department (ED) guideline and training protocol for PGY-1 residents on how to conduct a FAST exam, then upload and forward the bedside ultrasound scans in the hospital record system for review by Radiology.  Phase 1 of the project focused on baseline PACS system data collection and any associated reporting issues.

### METHODS

Phase 1 consisted of chart reviews for (1) PACS data (sonogram results) in charts that were activated as a trauma alert and (2) appropriate review by a radiologist. The chart review was conducted from July through September 2024.

### RESULTS

Of the 233 identified trauma cases requiring a FAST exam, only 2 FAST exams (1%) were properly uploaded and retrievable in the PACS system, with no exams (0%) being reviewed and verified by a radiologist.

### DISCUSSION/CONCLUSION

During the ACT segment of the Phase 1 PDSA QI cycle, baseline results were presented to the Director of the Emergency Department and Director of Trauma.  Discussions for obtaining new ultrasound software and equipment to improve the compliance of FAST examinations and uploading of the exams into the hospital record for appropriate review ensued from the presentation. These purchases will in turn enhance the treatment of trauma patients and improve patient safety and patient care. 

The results of this phase underscore the importance of (1) collecting accurate baseline data for any project, (2) the potential identification of issues that may have an indirect impact on a project’s outcome, and (3) the need to annotate potential confounding factors on results.

